# Evaluation of Machine Learning Algorithms for Classification of Visual Stimulation-Induced EEG Signals in 2D and 3D VR Videos

**DOI:** 10.3390/brainsci15010075

**Published:** 2025-01-16

**Authors:** Mingliang Zuo, Xiaoyu Chen, Li Sui

**Affiliations:** 1 School of Health Science and Engineering, University of Shanghai for Science and Technology, Shanghai 200093, China; 2 School of Information Science and Technology, Fudan University, Shanghai 200433, China

**Keywords:** machine learning, virtual reality, power spectral density, common spatial patterns, random forests

## Abstract

Backgrounds: Virtual reality (VR) has become a transformative technology with applications in gaming, education, healthcare, and psychotherapy. The subjective experiences in VR vary based on the virtual environment’s characteristics, and electroencephalography (EEG) is instrumental in assessing these differences. By analyzing EEG signals, researchers can explore the neural mechanisms underlying cognitive and emotional responses to VR stimuli. However, distinguishing EEG signals recorded by two-dimensional (2D) versus three-dimensional (3D) VR environments remains underexplored. Current research primarily utilizes power spectral density (PSD) features to differentiate between 2D and 3D VR conditions, but the potential of other feature parameters for enhanced discrimination is unclear. Additionally, the use of machine learning techniques to classify EEG signals from 2D and 3D VR using alternative features has not been thoroughly investigated, highlighting the need for further research to identify robust EEG features and effective classification methods. Methods: This study recorded EEG signals from participants exposed to 2D and 3D VR video stimuli to investigate the neural differences between these conditions. Key features extracted from the EEG data included PSD and common spatial patterns (CSPs), which capture frequency-domain and spatial-domain information, respectively. To evaluate classification performance, several classical machine learning algorithms were employed: ssupport vector machine (SVM), k-nearest neighbors (KNN), random forest (RF), naive Bayes, decision Tree, AdaBoost, and a voting classifier. The study systematically compared the classification performance of PSD and CSP features across these algorithms, providing a comprehensive analysis of their effectiveness in distinguishing EEG signals in response to 2D and 3D VR stimuli. Results: The study demonstrated that machine learning algorithms can effectively classify EEG signals recorded during watching 2D and 3D VR videos. CSP features outperformed PSD in classification accuracy, indicating their superior ability to capture EEG signals differences between the VR conditions. Among the machine learning algorithms, the Random Forest classifier achieved the highest accuracy at 95.02%, followed by KNN with 93.16% and SVM with 91.39%. The combination of CSP features with RF, KNN, and SVM consistently showed superior performance compared to other feature-algorithm combinations, underscoring the effectiveness of CSP and these algorithms in distinguishing EEG responses to different VR experiences. Conclusions: This study demonstrates that EEG signals recorded during watching 2D and 3D VR videos can be effectively classified using machine learning algorithms with extracted feature parameters. The findings highlight the superiority of CSP features over PSD in distinguishing EEG signals under different VR conditions, emphasizing CSP’s value in VR-induced EEG analysis. These results expand the application of feature-based machine learning methods in EEG studies and provide a foundation for future research into the brain cortical activity of VR experiences, supporting the broader use of machine learning in EEG-based analyses.

## 1. Introduction

Virtual Reality (VR) has become a groundbreaking technology with extensive applications across fields such as education, rehabilitation, and entertainment, fundamentally reshaping how individuals engage with digital environments. VR experiences are typically delivered in two primary formats: two-dimensional (2D) and three-dimensional (3D), each eliciting distinct subjective experiences. While 2D VR provides flat, non-interactive visuals, 3D VR offers dynamic, interactive environments that significantly enhance the sense of immersion. Studies such as Smith et al. (2021) suggest that 3D VR generates a stronger feeling of presence and engagement, as users can interact more deeply with virtual objects and settings, making it particularly effective in domains requiring high levels of immersion, such as education and therapy [1]. Furthermore, advancements in VR technology have led to the development of more realistic and customizable VR environments, impacting user engagement and cognitive load management [2,3].

Electroencephalography (EEG) has emerged as a critical tool for evaluating the subjective experiences elicited by various VR environments. Among the key analytical methods, power spectral density (PSD) measurements have proven to be especially valuable in assessing EEG signal variations under different VR conditions, such as 2D versus 3D environments. PSD analysis enables researchers to investigate changes in brain cortical activity across distinct frequency bands, including theta (4–8 Hz), alpha (8–12 Hz), and beta (13–30 Hz) [4]. Research by Huang et al. (2016) indicates that increased alpha band activity has been linked to enhanced cognitive performance and sustained attention during tasks requiring focus, while elevated beta band activity has been associated with improved motor control and more efficient information processing in complex scenarios [5]. Previous studies consistently demonstrate that PSD serves as a robust and reliable metric for capturing the brain cortical responses induced by VR stimuli, highlighting its importance in understanding the cognitive and emotional mechanisms underlying VR experiences [6].

Common spatial patterns (CSPs), a widely utilized feature extraction method in EEG-based brain–computer interface (BCI) research, has demonstrated superior performance in capturing the spatial characteristics of EEG signals compared to PSD. By effectively extracting spatial variance, CSP offers enhanced discriminatory power for differentiating brain cortical activity under varying conditions, making it particularly effective for identifying subtle differences in brain responses elicited by 2D versus 3D VR environments [7,8]. While CSP has shown considerable success in applications such as motor imagery classification in BCIs, its application to the analysis of VR-related EEG signals remains limited. Most current studies continue to rely predominantly on PSD features, which, although valuable, may fail to account for critical spatial information inherent in EEG signals [2]. This limited application of CSP represents a shortcoming in the literature. Leveraging CSP for the analysis of VR-related EEG data has the potential to provide deeper insights into the brain cortical mechanisms underlying VR-induced cognitive and emotional processes, while also contributing to advancements in VR-based BCI systems [9]. Consequently, further research is needed to explore and optimize the use of CSP in differentiating EEG signals across varying VR modalities, thereby advancing both the understanding of VR neuroscience and the development of EEG-based BCI technologies.

However, a broader review of existing methods reveals several limitations in the literature. For example, Zhang et al. (2020) employed deep learning methods with PSD features to classify engagement levels in VR tasks, yet did not compare these approaches against more classical machine learning models [2]. Similarly, Ghule et al. (2021) introduced wavelet-based features to study emotional states in an immersive VR environment, but focused on a single classification algorithm (SVM), limiting insights into how different models might handle VR-related EEG [10]. Moreover, many studies rely on narrowly defined feature sets (e.g., PSD alone) without leveraging spatial filters like CSP, or fail to examine multiple classification approaches in a unified experimental design [11,12]. As a result, PSD and CSP were chosen for this study owing to their complementary nature—PSD provides a frequency-based perspective, while CSP captures spatial distinctions across different VR conditions. This two-pronged approach aims to address a limitation noted in previous VR EEG research, where either PSD or CSP alone was predominantly used.

While machine learning algorithms have been extensively applied to EEG-based BCI research, their application to the classification of VR-related EEG signals, particularly under 2D and 3D VR conditions using feature-based approaches such as CSP, remains underexplored. Furthermore, there is a notable absence of comprehensive comparisons among multiple algorithms for classifying EEG signals recorded during watching 2D and 3D VR videos. For instance, Na et al. (2021) utilized KNN for classifying VR-related EEG but did not benchmark its performance against other algorithms like SVM or Random Forest [13]. Similarly, Guan et al. (2019) applied Decision Tree models to EEG data but did not explore how ensemble methods like AdaBoost could improve performance in VR contexts [8]. These limitations in the literature highlight the need for more systematic evaluations that incorporate frequency and spatial feature extraction methods, along with a range of machine learning classifiers, enabling a deeper understanding of how different approaches perform across various scenarios.

To address these limitations, the present study employed multiple machine learning algorithms to evaluate the classification performance of EEG signals based on two feature extraction methods: PSD and CSP. EEG data were collected during exposure to 2D and 3D VR video, and the extracted features were used to train and test various machine learning models, including support vector machine (SVM) [14], k-nearest neighbors (KNN) [13], random forest (RF) [7], naive Bayes [15], decision tree (DT) [8], AdaBoost [16], and a voting classifier [17]. The classification performance of these models was assessed using metrics such as accuracy, precision, recall, and F1-score, providing a comprehensive comparison of their effectiveness in distinguishing between EEG signals recorded during watching 2D and 3D VR videos. This study offers valuable insights into the potential of feature-based machine learning approaches for analyzing VR-related EEG data and highlights the importance of systematic evaluations across diverse algorithms.

## 2. Materials and Methods

### 2.1. Subjects

The experiment comprised 20 healthy subjects (10 women and 10 men) from the University of Shanghai for Science and Technology, with a mean age of 23.8 ± 1.45 years. All participants had normal or corrected vision, were in good physical health, demonstrated standard stereoscopic vision, and reported no history of neurological disorders, other illnesses, or symptoms of sickness. None of the subjects had a mental illness affecting cognitive ability, and all had prior experience with VR videos. Additionally, they were not undergoing any medical treatment that could impact cognitive functioning or proprioceptive systems.

All experimental procedures complied with the Declaration of Helsinki and adhered to the ethical standards of the local institution [18]. Participants were informed of their right to withdraw from the experiment at any time, and written informed consent was obtained from all participants. The study was approved by the Ethics Committee of the University of Shanghai for Science and Technology.

### 2.2. EEG Acquisition

**Figure 1:**
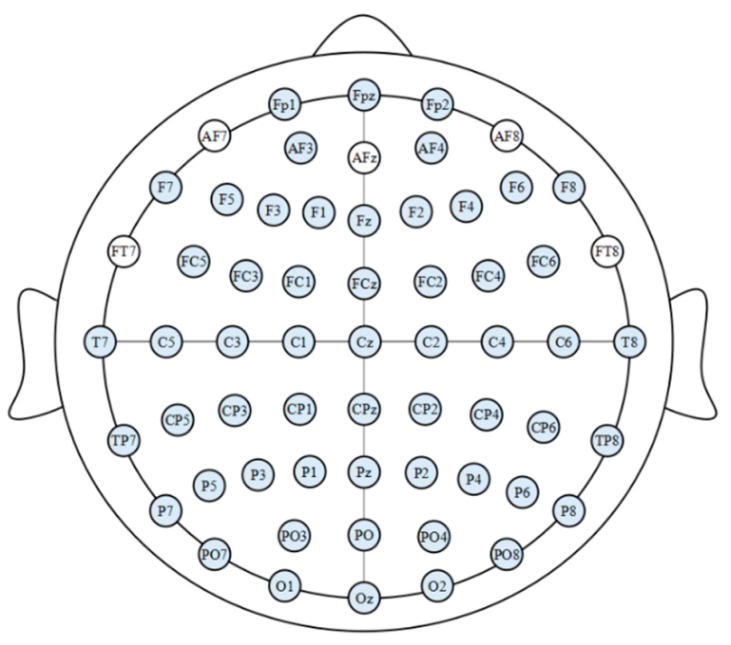
Electrode lead position layout.

The experiment employed an iPhone XR with a 5.5-inch, 16:9 screen, Google Cardboard VR glasses, and the VR application. EEG data was acquired using the Grael EEG amplifier (Compumedics Neuroscan), a 2017 model capable of simultaneous wireless data collection from multiple participants. Silver/AgCl electrodes were positioned according to the 10–20 international system using an EEG cap (Compumedics Neuroscan). The experimenter configured a montage of 56 active electrodes in the 10–20 system. We selected a montage of 56 active electrodes according to the 10–20 system and used the EEG signals recorded from these sites for the study. The specific positions and names of these electrode locations, highlighted against a blue background in Figure 1, are detailed for clarity.

**Figure 2:**
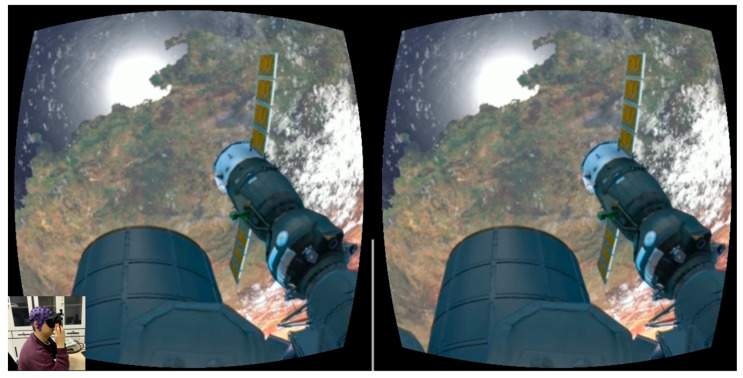
A space VR scene in a spacecraft, showing a participant in the lower-left corner of the image wearing an EEG cap and VR glasses while watching VR videos in an isolated room.

Subjects were seated in comfortable chairs within isolated rooms, equipped with EEG caps and Google Cardboard VR glasses, while viewing VR videos. The experiment aimed to record EEG signals recorded in response to the stimuli from both 2D and 3D VR videos. Twenty healthy participants took part in the study, with each participant viewing four 5 min 2D videos and four 5 min 3D videos. Both 2D and 3D videos featured identical scenes designed to evoke various environments, including snow-covered landscapes, oceanic vistas, dense forests, and outer space. These virtual environments were carefully selected to ensure consistency across all participants, minimizing potential confounding effects from differences in visual stimuli. The scenes in both video formats were identical in content and structure, meaning all participants watched the same sequences of videos. To prevent interference from watching different videos, participants were allowed a 5–10 min rest period after each video to help maintain focus and avoid fatigue affecting the results. Figure 2 illustrates a typical Space VR scene set within a spacecraft, one of the environments used in the experiment. In the lower-left corner of the image, a participant is shown wearing an EEG cap and VR glasses while engaging with the VR videos in an isolated room. This controlled setup ensured that participants’ attention was focused entirely on the video stimuli without external distractions.

### 2.3. EEG Processing

EEG data processing was conducted using Curry software (Compumedics Neuroscan), version [9.0]. For each participant, 300 s of continuous EEG data was recorded at an original sampling rate of 1024 Hz. The EEG signals were initially re-referenced to the average of all electrodes, including the original reference electrode, to ensure the data retained full rank [19].

A 4th-order Butterworth bandpass filter with a frequency range of 0.5–40 Hz was applied to eliminate low-frequency drifts and high-frequency noise, using a Hamming window function with a transition band of 0.3–0.5 Hz at the low end and 40–42 Hz at the high end. Following bandpass filtering, a Notch filter with a frequency range of 48–52 Hz was applied to remove 50 Hz power-line interference from the EEG signal. Subsequently, the data were downsampled to 512 Hz to improve computational efficiency while maintaining the Nyquist criterion.

Time segments containing significant signal drift caused by participant movements or other artifacts were identified and manually removed using Curry’s interactive graphical interface. These segments were visually inspected, marked, and excluded from further analysis using the REJECT function to ensure high-quality data for subsequent processing.

The ICA algorithm implemented in Curry software was then applied to decompose the EEG signals into independent components, effectively separating brain cortical signals from artifacts [20]. The ICA decomposition was performed using the Infomax algorithm with the default parameters provided by the Curry software. Each independent component was inspected visually, focusing on the scalp topographies, power spectral densities, and temporal dynamics of the components to identify artifacts. Artifact-related components were identified based on specific patterns, such as frontal high-amplitude activity for eye blinks, lateralized activity for horizontal eye movements, high-frequency power for muscle artifacts, and irregular patterns for noisy electrodes. Components identified as artifact-related were excluded by selecting the REJECT option, while non-artifact components were retained to reconstruct the cleaned EEG signals. This process ensured that the final EEG data were free of significant artifacts while preserving the brain cortical signal integrity.

To facilitate further analysis, the continuous EEG signals were segmented into 2 s intervals [21]. This segmentation allowed for the efficient analysis of shorter, stable data segments while avoiding the computational challenges of processing the entire continuous recording.

All EEG channels were retained as none exhibited poor signal quality during the experiment. Signal quality was assessed through visual inspection, checking for amplitude abnormalities, flatline signals, or excessive noise. Time segments with significant signal drift or other anomalies were excluded as described above, ensuring the remaining dataset was of high quality for subsequent analysis without the need for channel interpolation.

**Figure 3:**
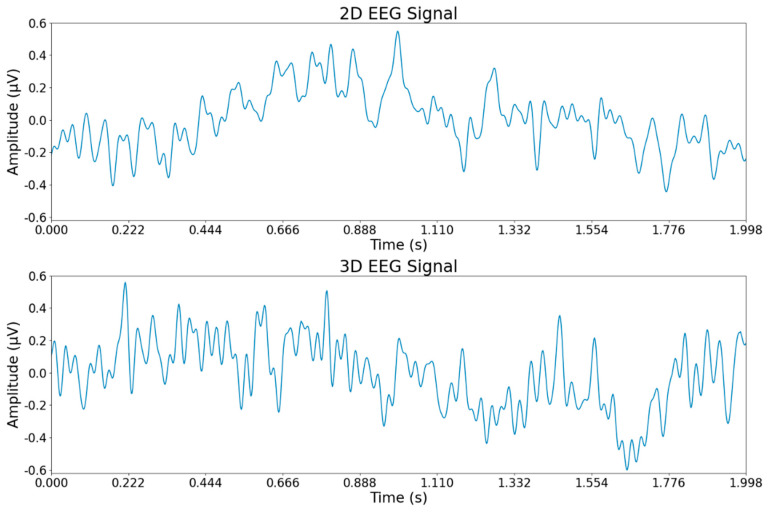
EEG signals from the O2 channel, recorded during the first 2 s of both 2D and 3D VR video viewing, are displayed with the 2D signals at the top and the 3D signals at the bottom.

The processed EEG dataset was saved in CNT format, with each 300 s experiment utilizing 56 channels and sampled at 512 Hz. The continuous recordings were segmented into 2 s intervals using an epoch object, resulting in a three-dimensional array of dimensions ($n_{epochs}$, $n_{channels}$, $n_{times}$), where $n_{channels}$ denotes the number of channels and $n_{times}$ represents the number of time points per epoch ($cycle=\frac{1}{sampling\;rate}$). Figure 3 depicts the EEG signals from a sample channel (e.g., O2) for the first 2 s of the dataset. The top section shows the EEG signals recorded while participants watched 2D videos, and the bottom section illustrates the corresponding signals recorded during 3D video viewing for the same channel and time period.

### 2.4. Feature Extraction

The choice of PSD and CSP as feature extraction methods in this study was guided by their effectiveness in analyzing EEG signals for VR-related tasks. PSD was selected for its ability to quantify the power distribution across distinct frequency bands, which are closely associated with cognitive and emotional states. Specifically, the alpha band (8–12 Hz) reflects relaxation and sustained attention, while the beta band (13–30 Hz) correlates with motor control and cognitive engagement [22,23]. By analyzing these bands, PSD provides interpretable frequency-domain features that are essential for understanding neural activity during 2D and 3D VR exposure.

CSP was chosen due to its proven capability in capturing spatial variance across multiple EEG channels, which is critical for distinguishing subtle neural differences induced by immersive VR environments. CSP emphasizes variance differences between classes, enhancing class separability, and has demonstrated good performance in brain–computer interface (BCI) research, particularly for motor imagery classification and workload monitoring tasks [24,25]. Given that spatial patterns play a crucial role in VR-related EEG analysis, CSP was particularly suitable for this study.

#### 2.4.1. Power Spectral Density

The PSD measurements were extracted from artifact-free EEG epochs using the Welch method across all 56 electrode sites [4].

The initial definition of the PSD is derived using the Fourier Transform, as described in Formula (1).

(1) $$S_{xx}\left(f\right)=\underset{T\to \infty }{\lim}\frac{1}{T}{\left|X_{T}\left(f\right)\right|}^{2}$$

where $X_{T}\left(f\right)=\int_{-T/2}^{T/2}x\left(t\right)e^{-j2\pi f\tau }d\tau$ is the Fourier Transform of the signal $x\left(t\right)$ over the time interval T, and f represents frequency. Formula (2) represents the Wiener–Khinchin theorem, which relates the PSD to the autocorrelation function $R_{xx}$.

(2) $$S_{xx}\left(f\right)=\int_{-\infty }^{\infty }R_{xx}\left(\tau \right)e^{-j2\pi f\tau }d\tau$$

Here, $R_{xx}\left(\tau \right)=E\left[x\left(t\right)x\left(t+\tau \right)\right]$ is the autocorrelation function, where E denotes the expected value and τ is the time lag.

**Figure 4:**
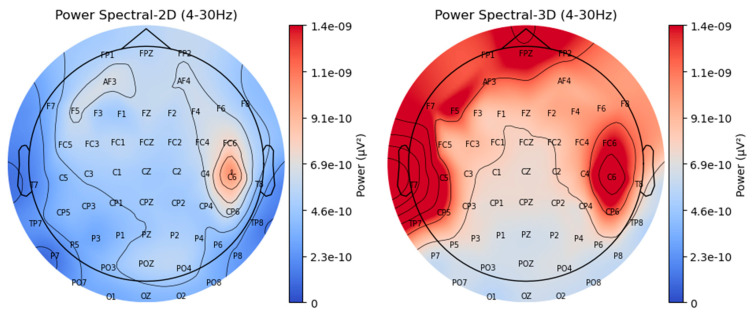
Power spectral maps: 2D (**left**) and 3D (**right**).

Figure 4 demonstrates the differences in the power spectrum obtained during 2D and 3D VR experiments. As shown in the figure, power spectral maps visualize power levels using color intensity: warmer colors (e.g., reds) indicate higher power, while cooler colors (e.g., blues) indicate lower power. The data presented in Figure 4 were aggregated across all participants, representing the average power spectrum for each condition. Individual variation in EEG responses was not analyzed separately in this study, as the focus was on identifying general trends across participants. The map on the left illustrates the power distribution across the scalp for 2D data within the 4–30 Hz frequency range, while the map on the right shows the corresponding distribution for 3D data in the same frequency range. In the 2D VR condition, the most prominent activity is observed at channels C6, AF3, and F5, highlighting these as key regions of engagement. In contrast, the 3D VR condition demonstrates a substantial increase in power across multiple electrode sites, with pronounced activity in the frontal, left lateral, and right lateral regions compared to 2D VR.

#### 2.4.2. Common Spatial Patterns

For CSP measurements, spatial filters are derived through generalized eigenvalue decomposition, transforming multi-channel EEG data into a new space to enhance class separability. CSP emphasizes the variance between two classes by projecting the EEG signals accordingly. The variance of these transformed signals serves as a key feature for subsequent classification, making CSP an essential measurement technique for classifying EEG signals in both 2D and 3D VR environments.

To derive CSP filters using generalized eigenvalue decomposition, Equation (3) is solved.

(3) $$R_{1}w=\lambda R_{2}w$$

where $R_{1}$ and $R_{2}$ are the covariance matrices of the two classes, w represents the spatial filters, and λ represents the eigenvalues. The eigenvectors w correspond to spatial filters that maximize variance differences between classes. To calculate the variance of the transformed signal and obtain the spatial filter matrix W, it is applied to the EEG data X to obtain the transformed signal Z as shown in Equation (4).

(4) $$Z=W^{T}X$$

The variance of Z is then used as a key feature for distinguishing between classes.

**Figure 5:**
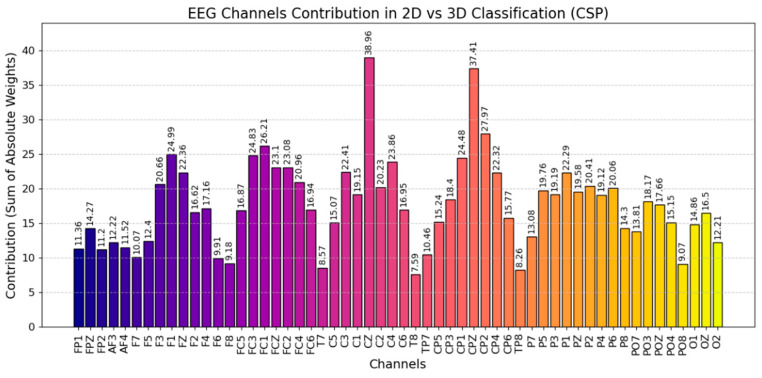
The contribution of various EEG channels in classification using CSP feature extraction.

Figure 5 shows the contribution of various EEG channels in classification using CSP feature extraction. The *X*-axis lists the EEG channels, while the *Y*-axis displays the contribution values. Higher values indicate a greater significance of that channel in the classification. Channels such as C2 (38.96), CPZ (37.41), and CP2 (27.97) have higher weights, indicating they contribute significantly to the variance differences captured by CSP filters in this classification task. This indicates that these channels are particularly sensitive to EEG signals, which help classify EEG data from both 2D and 3D VR environments. Lower-weighted channels, such as T8 (7.59), TP8 (8.26), and T7 (8.57), contribute less, suggesting they capture less variance related to the classification and may be less relevant.

### 2.5. Machine Learning Classification

#### 2.5.1. Support Vector Machine

The goal of a support vector machine (SVM) is to find the optimal hyperplane that separates the data points of different classes with the maximum margin. The decision function of an SVM can be represented mathematically as Equation (5); this margin is maximized, ensuring that the hyperplane is positioned in a way that best separates the classes.

(5) $$f\left(x\right)=w\cdot x+b$$

where w is the weight vector, x is the input vector, and b is the bias term. Equations (6) and (7) ensure that the hyperplane is not only correctly classifying the data points but also doing so with the maximum possible margin, leading to better generalization on unseen data. $y_{i}$ represents the class label of the i training sample, which can be either +1 or −1, and $x_{i}$ is the i training sample.

(6) $${min}_{w,b}\frac{1}{2}{\left||w|\right|}^{2}$$

(7) $$y_{i}\left(w\cdot x_{i}+b\right)\geq 1$$

#### 2.5.2. K-Nearest Neighbors

K-nearest neighbors (KNN) is a simple, non-parametric, and lazy learning algorithm used for classification and regression tasks. It operates on the principle that similar data points are likely to be close to each other. The algorithm classifies a data point based on the majority class among its k-nearest neighbors in the feature space. The core operation of the KNN algorithm is to measure similarity through a distance calculation (Equation (8)), where $x_{i}$ and $x_{j}$ are two data points, and $x_{ik}$ and $x_{jk}$ are the k features of these data points.

(8) $$d\left(x_{i},x_{j}\right)=\sqrt{\sum_{k=1}^{n}{\left(x_{ik}-x_{jk}\right)}^{2}}$$

Decisions are made based on the majority class of the nearest neighbors (Equation (9)), where $y_{i}$ represents the class of the i neighbor and the predicted class is $\hat{y}$.

(9) $$\hat{y}=mode\left(y_{1},y_{2},\cdot \cdot \cdot ,y_{k}\right)$$

#### 2.5.3. Random Forest

**Figure 6:**
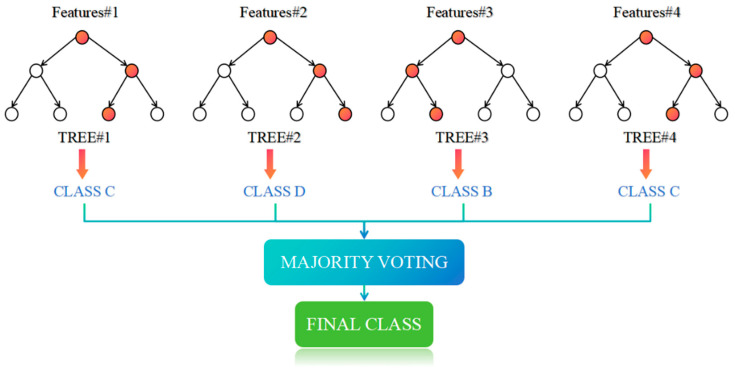
Architectural structure of the RF algorithm.

A random forest (RF) operates by constructing a multitude of decision trees during training and outputs either the mode of the classes for classification or the mean prediction for regression from the individual trees, with its architectural structure illustrated in Figure 6. Gini impurity for classification is shown in Equation (10), where n is the number of classes and $p_{i}$ is the probability of an element being classified into a particular class.

(10) $$Gini\left(p\right)=1-\sum_{i-1}^{n}p_{i}^{2}$$

The mean squared error for regression is shown in Equation (11), where $y_{i}$ is the actual value and ${\hat{y}}_{i}$ is the predicted value by the model.

(11) $$MSE=\frac{1}{n}\sum_{i-1}^{n}\left(y_{i}-{\hat{y}}_{i}\right)$$

#### 2.5.4. Logistic Regression

Logistic Regression (LR) models the probability that a given input belongs to a particular class, which predicts the probability of a binary outcome. The logistic function (Equation (12)) models the probability of class membership, where z=w·x+b, with w being the weight vector, x being the input vector, and b being the bias term.

(12) $$\sigma \left(z\right)=\frac{1}{1+e^{-z}}$$

Additionally, the log-likelihood function is used to estimate the parameters that best fit the data, where $y_{i}$ is the actual label (0 or 1) for the i sample, ${\hat{y}}_{i}=\sigma \left(w\cdot x_{i}+b\right)$ is the predicted probability for the i-th sample, and n is the number of samples, as shown in Equation (13).

(13) $$\left(w,\;b\right)=\sum_{i=1}^{n}\left[y_{i}\log\left({\hat{y}}_{i}\right)+\left(1-y_{i}\right)\log\left(1-{\hat{y}}_{i}\right)\right]$$

#### 2.5.5. Gaussian Naive Bayes

Gaussian naive Bayes (GNB) is a variant of the naive Bayes classifier that assumes the data features follow a Gaussian (normal) distribution. It estimates the probability of each class for a given set of features and then classifies the instance into the class with the highest probability. The foundation of naive Bayes is Bayes’ theorem, which is used to calculate the posterior probability P(X|C)·P(C), or the probability of a class C given the feature vector X. The formula is expressed as Equation (14):

(14) $$P\left(C|X\right)=\frac{P\left(X|C\right)\cdot P\left(C\right)}{P\left(X\right)}$$

P(C|X) is the posterior probability of class C given the feature vector X, P(X|C) is the likelihood of X given class C, P(C) is the prior probability of class C, and P(X) is the marginal probability of the feature vector X. Among them, the Gaussian likelihood formula expressed as Equation (15):

(15) $$\left(x_{i}|C\right)=\frac{1}{\sqrt{2\pi \sigma_{C}^{2}}}exp\left(-\frac{{\left(x_{i}-\mu_{C}\right)}^{2}}{2\sigma_{C}^{2}}\right)$$

This formula represents the probability of observing a feature value $x_{i}$ given that the data point belongs to class C. GNB assumes that each feature follows a Gaussian distribution for each class, and it estimates the parameters $\mu_{C}$ and $\sigma_{C}$ during training.

#### 2.5.6. Decision Tree

A decision tree works by recursively splitting the data into subsets based on the feature that provides the best separation, creating a tree-like structure of decisions. Each internal node represents a decision based on a feature, each branch represents the outcome of that decision, and each leaf node represents a class label (in classification) or a numerical value (in regression). Gini impurity measures the probability of misclassifying a randomly chosen element from the set if it were labeled according to the distribution of labels in the subset. For a given node, Gini impurity is calculated as in Equation (16). Information Gain represents the reduction in uncertainty about the class labels when splitting the data according to a particular attribute.

(16) $$Information\;Gain=Entropy\left(D\right)-\sum_{j=1}^{k}\frac{\left|D_{j}\right|}{\left|D\right|}\;Entropy\left(D_{j}\right)$$

where D is the original dataset, and $D_{j}$ represents the subsets resulting from the split. Higher Information Gain indicates a more informative feature for the split.

#### 2.5.7. Adaptive Boosting

Adaptive boosting, commonly known as AdaBoost, combines multiple weak classifiers to form a strong classifier with improved predictive performance. At each iteration t, AdaBoost updates the weights of the training samples based on the performance of the weak classifier $h_{t}$. The weight update formula for each sample i is shown in Equation (17).

(17) $$D_{t+1}\left(i\right)=\frac{D_{t}\left(i\right)\times e^{-\alpha_{t}y_{i}h_{t}\left(x_{i}\right)}}{Z_{t}}$$

In this formula, $D_{t}\left(i\right)$ represents the weight of sample i at iteration t, $y_{i}$ is the true label of sample i, $h_{t}\left(x_{i}\right)$ is the prediction of the weak classifier for sample i, $\alpha_{t}$ is the weight assigned to the weak classifier based on its performance, and $Z_{t}$ is a normalization factor ensuring that the sum of the weights $D_{t+1}$ equals 1.

#### 2.5.8. Voting Classifier

A voting classifier combines the predictions of multiple diverse classifiers to improve overall performance and robustness. By aggregating the outputs of seven algorithms—SVM, KNN, random forests, logistic regression, naive Bayes, decision trees, and AdaBoost—the voting classifier leverages their individual strengths and mitigates their weaknesses, leading to better generalization on unseen data. The soft voting method averages the predicted class probabilities and selects the class with the highest average probability. The final predicted class $\hat{y}$ is determined by majority vote among all classifiers, as shown in Equation (18).

(18) $$\hat{y}=arg\;\underset{k}{\max}\left(\frac{1}{T}\sum_{t=1}^{T}P_{t}\left(y=k|x\right)\right)$$

where $P_{t}\left(y=k|x\right)$ is the predicted probability of class k from the t−th classifier. By integrating different models through voting, the voting classifier often achieves better predictive performance than any single constituent model, especially when the individual models are diverse and uncorrelated.

#### 2.5.9. Classification Pipeline

**Figure 7:**
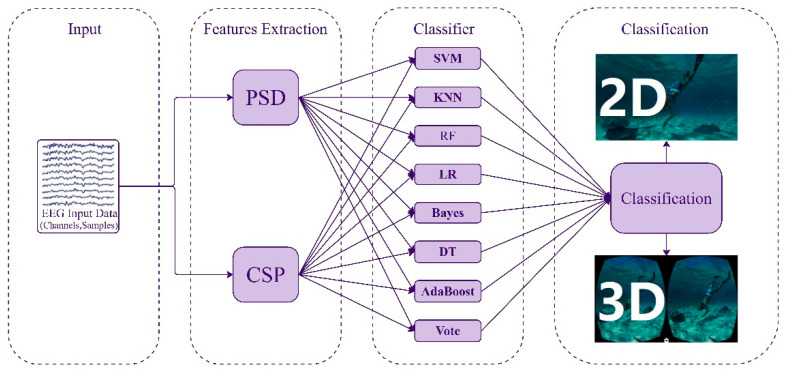
The classification pipeline includes PSD and CSP measures, normalization, and machine learning classification.

The proposed pipeline for computing and selecting features to train classifiers, followed by testing them using EEG datasets from a 2D/3D VR environment, is illustrated in Figure 7.

The classification pipeline begins with EEG data recorded during 2D and 3D VR experiences, which are preprocessed and then analyzed using two feature extraction methods: PSD for capturing frequency-based features, and CSP for identifying spatial patterns in brain signals. The resulting features are fed into multiple classifiers: SVM for separating hyperplanes [26], KNN for majority class classification, RF with decision tree ensembles, LR for binary classification, a Bayesian classifier for probabilistic methods, DT for rule-based decisions, AdaBoost for combining weak classifiers, and a voting ensemble to aggregate predictions from all classifiers for greater reliability. The study examined these eight machine learning models, which utilized PSD and CSP features input into SVM [26], KNN [13], RF [7], LR [27], Bayes [15], DT [8], AdaBoost [16], and a Voting Classifier [17] using Python’s scikit-learn library. The classification experiments were conducted in two main phases: training with data from seven segments and testing with data from three segments, while employing a repeated experiments approach in which each model was trained and tested 10 times with different random data splits. The final classification step determines whether the EEG data correspond to a 2D or 3D VR environment, allowing for a comparative analysis of brain activity across these experiences.

### 2.6. Validation Procedure and Performance Metrics

The classification experiments consisted of two main phases: training and testing, with models trained on data from seven segments and evaluated on data from three segments [28]. To ensure the reliability and robustness of the results, a repeated experiments method was employed. In this method, each model was trained and tested 10 times with different random splits of the data to minimize the impact of any single data split on the outcomes. Key metrics, including accuracy, precision, recall, and F1-score, were calculated for each run, and their mean values were used to summarize overall performance. To evaluate the variability and stability of the models, the standard deviation of these metrics was also computed across the 10 runs. The final results, showed as mean ± standard deviation, provide a detailed and reliable evaluation of each model’s classification capabilities. Brief explanations of these metrics are provided below:

Accuracy is a fundamental metric used in evaluating the performance of classification models [29]. Essentially, accuracy is a measure of how often the model is correct in its decision-making process. Mathematically, it is represented as in Equation (19).

(19) $$Accuracy=\frac{TP+TN}{TP+FP+TN+FN}$$

The formula for accuracy is expressed as the ratio of true positives (*TP*) and true negatives (*TN*) to the total number of predictions, which also includes false positives (*FP*) and false negatives (*FN*).

Precision specifically measures the accuracy of the model’s positive predictions [30]. In other words, it reflects how many of the instances that the model predicted as positive are actually positive. The formula for precision is expressed as in Equation (20).

(20) $$Precision=\frac{TP}{TP+FP}$$

Recall, also known as sensitivity or the hit rate, measures a model’s ability to correctly identify all relevant positive instances within a dataset [31]. The formula for recall is in Equation (21).

(21) $$Recall=\frac{TP}{TP+FN}$$

The *F*1-score offers a balanced evaluation of a model’s performance by both precision and recall. It is defined as the harmonic mean of precision and recall, with its formula presented in Equation (22).

(22) $$F1-score=2\times \frac{Precision\times Recall}{Precision+Recall}$$

### 2.7. Hardware and Software Configuration for the Experiment

The hardware environment for the experiments consisted of a computer featuring an Intel Core i5 processor, with a base clock speed of 2.5 GHz and the ability to boost up to 2.59 GHz. It was equipped with an NVIDIA GeForce RTX 4090 GPU, manufactured by NVIDIA Corporation, based in Santa Clara, California, USA, for high-performance graphical processing and 16 GB of RAM to ensure efficient data handling.

The software environment included Microsoft Windows 10 as the operating system, along with essential tools for the experiment. EEG data preprocessing and analysis were performed using Curry software (Compumedics Neuroscan). Feature extraction involved spectral features such as PSD and spatial features using CSP, implemented in Python (version 3.8) with libraries such as NumPy, pandas, and MNE. Machine learning models were developed and implemented using scikit-learn (version 1.4). Table 1 summarizes the key parameters for each classification model used in the study.

## 3. Results

In this classification experiment, we evaluated several machine learning models on our dataset by using combinations of PSD and CSP features as inputs to classifiers, including SVM [26], KNN [13], RF [7], LR [27], Bayes [15], DT [8], AdaBoost [16], and a voting classifier [17]. Each model was trained on data from seven parts of the dataset and tested on data from the remaining three parts while employing a repeated experiments approach in which each model was trained and tested 10 times with different random data splits.

**Figure 8:**
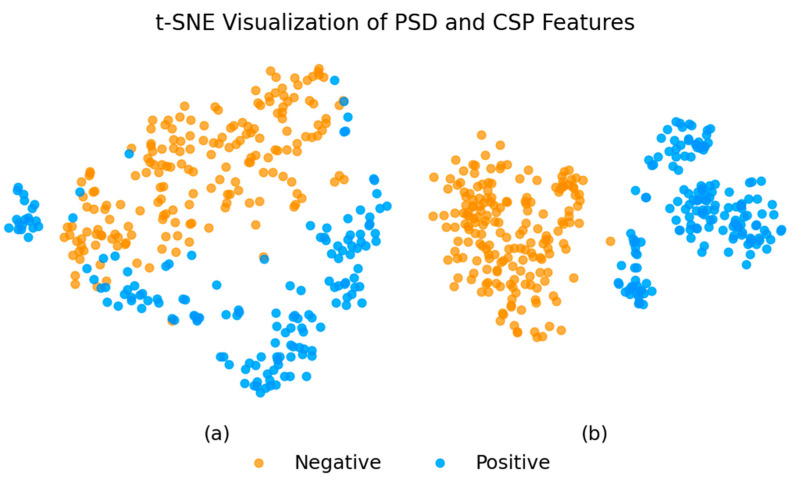
t-SNE visualization of features extracted using PSD (**a**) and CSP (**b**) methods.

Figure 8 shows the t-SNE visualization, illustrating that CSP features outperform PSD features in classifying EEG signals recorded during exposure to 2D/3D VR environments. PSD features result in a highly overlapping and scattered representation, leading to ambiguities that make accurate classification challenging. On the other hand, CSP features yield well-separated clusters, making it easier for classifiers to distinguish between positive and negative cases. The clear distinction between classes with CSP features results in better model performance, with fewer misclassifications and more reliable predictions.

**Figure 9:**
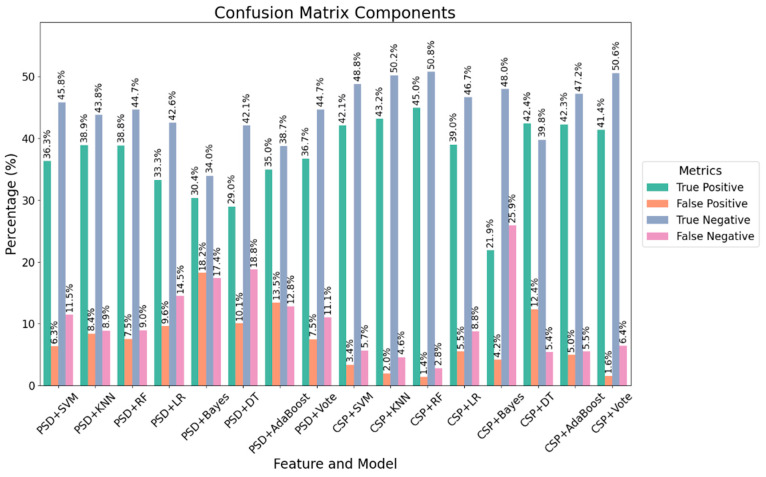
The mean confusion matrix components for all subjects using machine learning approaches.

Figure 9 shows the mean confusion matrix components for all machine learning approaches across all subjects. The CSP + RF combination is the optimal choice for the given dataset, providing the best balance of true positive and true negative rates while maintaining low false positive and false negative rates. CSP + KNN is another strong candidate, although it slightly lags behind CSP + RF. Overall, CSP features seem to offer more valuable information compared to PSD features, as they yield higher accuracy across multiple classifiers. The choice of classifier also significantly impacts performance, with random forest being the top performer in this analysis.

**Figure 10:**
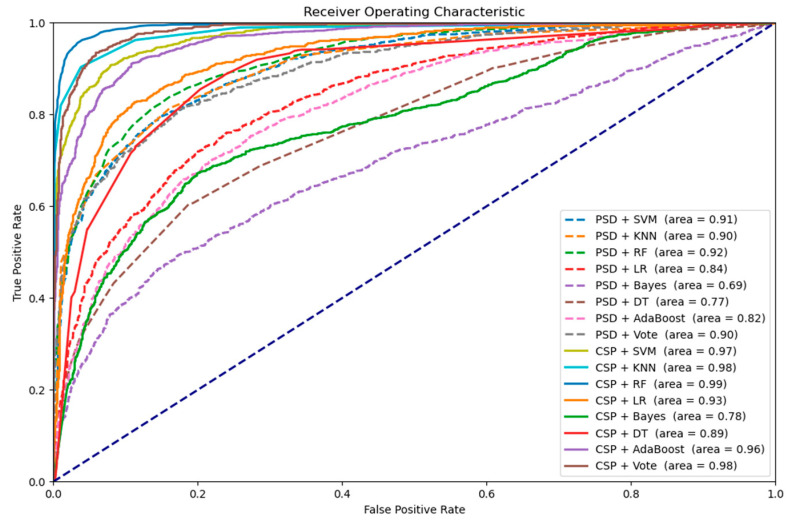
The ROC curves using machine learning approaches.

Figure 10 shows the receiver operating characteristic (ROC) curves generated by machine learning methods across all subjects, using multiple combinations of feature extraction and classification methods. Each curve compares the true positive rate to the false positive rate at different classification thresholds, with the area under the curve (AUC) indicating overall performance. Among the tested combinations, CSP + RF achieves the highest AUC, showing good performance in distinguishing EEG signals recorded during watching 2D/3D VR videos.

**Figure 11:**
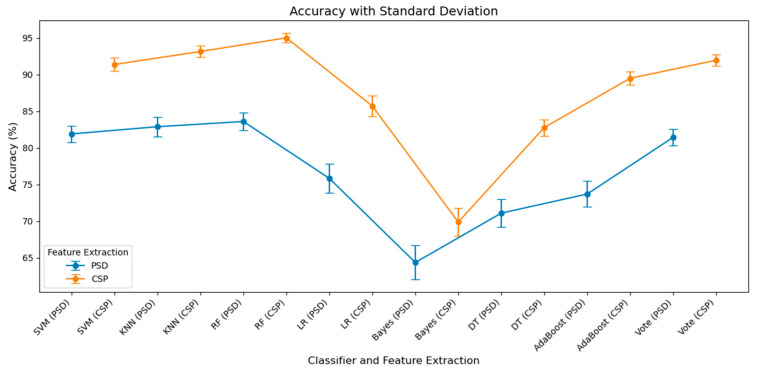
The accuracy with standard deviation curves using machine learning approaches.

Table 2 presents the outcomes for key performance indicators: accuracy, precision, recall, and F1-score for each classification method. Figure 11 illustrates the accuracy curves with standard deviations, providing a visual representation of the performance stability of different machine learning approaches. Overall, RF, KNN, and SVM emerged as the top-performing classifiers in this study, particularly when combined with CSP features, achieving accuracies of 95.02 ± 0.67%, 93.16 ± 0.76%, and 91.39 ± 0.89%, respectively. LR, DT, and the voting classifier also showed notable improvements when using CSP features, with accuracies of 85.71 ± 1.45%, 82.78 ± 1.12%, and 91.96 ± 0.75%, respectively. Random forest demonstrated the most balanced performance across all metrics, achieving an F1-score of 94.48 ± 0.75%, highlighting its strong classification capabilities. Additionally, KNN and the voting classifier showed excellent precision, reaching 94.35 ± 0.89% and 96.27 ± 0.68%, respectively, indicating their effectiveness in minimizing misclassification. While naive Bayes and AdaBoost exhibited relatively weaker performance, they still showed improvement when CSP features were utilized.

## 4. Discussion

In this study, EEG data were collected from 20 participants as they viewed less immersive 2D VR videos and fully immersive 3D VR videos. The primary goal was to classify EEG signals corresponding to these two distinct VR experiences. To achieve this, a range of machine learning classifiers and feature extraction methods were evaluated to identify the combinations that yielded the best classification performance.

The results revealed that RF, KNN, and SVM, when combined with CSP features, achieved the highest classification accuracies. Among these, RF emerged as the top-performing model, achieving an accuracy of 95.02 ± 0.67%, a precision of 95.15 ± 0.82%, and an F1-score of 94.48 ± 0.75%. These findings align with previous studies demonstrating the effectiveness of ensemble methods like RF for handling high-dimensional EEG data [1,32]. For instance, Fang et al. (2020) observed that RF consistently outperformed single-tree models like decision tree (DT) in EEG classification tasks due to its ability to mitigate overfitting and enhance robustness through ensemble learning [6]. Similarly, RF’s capability to aggregate multiple decision trees allowed it to capture diverse patterns in EEG data, a critical advantage when working with complex signals such as those recorded during VR experiences [33].

KNN also demonstrated performance well, with an accuracy of 93.16 ± 0.76%, likely benefiting from the clear separation between classes provided by CSP features. The proximity-based classification approach of KNN is particularly effective for distinguishing subtle variations in EEG signals, as shown in prior studies on motor imagery tasks and VR classification [34,35]. Sharmila and Geethanjali (2016) highlighted the advantages of KNN in preserving the integrity of high-dimensional EEG features, which aligns with the performance observed in this study [36]. Similarly, SVM, which achieved an accuracy of 91.39 ± 0.89%, leveraged its ability to find optimal hyperplanes that maximize class separation. This result is consistent with findings by Ma et al. (2016), who demonstrated SVM’s effectiveness in distinguishing EEG patterns under different task conditions, particularly when combined with spatially enhanced features like CSP [13].

In contrast, classifiers such as logistic regression (LR), naive Bayes, DT, AdaBoost, and the voting classifier generally underperformed compared to RF, KNN, and SVM. For example, the linear assumptions of LR limited its ability to capture the non-linear relationships inherent in EEG data, as noted in Prabhakar et al. (2020) [37]. Similarly, naive Bayes, which assumes feature independence, struggled to model the interdependent nature of EEG features effectively. This aligns with the findings, which highlighted naive Bayes’ limitations in handling correlated EEG features [38]. DT models were prone to overfitting, particularly given the relatively small dataset size, a challenge previously reported in EEG studies by Guan et al. (2019) [8]. Although AdaBoost is designed to improve the performance of weak classifiers, its reliance on these base classifiers may have limited its ability to outperform RF’s ensemble strategy, as also observed by Kamble and Sengupta (2021) [9].

The standard deviation values in Table 2 provide key insights into the stability and reliability of the classifiers. Models such as RF, KNN, and SVM demonstrated the most stable performances, with standard deviations consistently below 1% across all metrics when using CSP features, indicating reliable and consistent performance across repeated experiments. For example, RF achieved an accuracy of 95.02 ± 0.67%, KNN reached 93.16 ± 0.76%, and SVM achieved 91.39 ± 0.89%. In contrast, classifiers like naive Bayes and LR exhibited higher standard deviations, reflecting greater variability and reduced reliability. Naive Bayes, for instance, had an accuracy of 69.87 ± 1.89% and a recall of 45.78 ± 2.32%, while LR showed a standard deviation of ±1.45% for accuracy, indicating more fluctuation in performance. These results highlight that models with lower standard deviations, such as RF, KNN, and SVM, demonstrate greater stability.

Despite these promising results, this study has several limitations. One major limitation is the use of binary classification to distinguish EEG signals recorded during 2D and 3D VR experiences. This approach simplifies the diversity of VR experiences into two categories, failing to capture the nuances of participants’ interactions with immersive content. Factors such as content relevance, degree of realism, and participants’ personal interests can significantly influence VR engagement levels, yet these factors were not fully accounted for in this study. This aligns with findings by Marta et al. (2024), who emphasized the need for more granular EEG analyses that consider individual differences in VR engagement [34]. Additionally, the study relied solely on PSD and CSP measures for feature extraction, without exploring other potential features such as entropy measures or functional connectivity metrics. For instance, studies like Aziz et al. (2018) and Yu et al. (2022) have demonstrated the potential of entropy-based features and graph-theoretical measures to enhance EEG classification in immersive settings [6,39]. Incorporating these additional features could improve classification performance and provide deeper insights into the neural mechanisms underlying VR experiences. While this study demonstrates the effectiveness of CSP features in classifying EEG signals, the findings should be interpreted in light of certain limitations. CSP focuses on optimizing spatial variance and may not capture frequency-domain information as effectively as PSD. Alternative methods, such as entropy-based features (e.g., sample entropy, permutation entropy), wavelet transforms, or graph-theoretical measures, could provide complementary insights into the complexity and connectivity of EEG signals [40,41]. For instance, entropy measures have been shown to capture the non-linear dynamics of brain activity, which could enhance classification accuracy in scenarios involving complex cognitive tasks such as VR immersion [42]. Although this study focused on PSD and CSP, future work should explore entropy measures, wavelet transforms, and functional connectivity metrics to enhance feature extraction. These methods could provide complementary insights into the neural mechanisms underlying VR-induced cognitive and emotional processes, addressing the potential limitations of frequency- and spatial-domain features alone.

Furthermore, while the current evaluation methodology involved training on seven segments and testing on three, it does not incorporate cross-validation. Instead, we followed a standard 70–30% training–test split, ensuring that the training data are separate from the test data, thereby avoiding contamination. However, the lack of cross-validation represents a methodological limitation. Techniques such as k-fold cross-validation could provide a more robust evaluation by offering additional insight into model performance variability across different data splits, as suggested by Ünalan, Serhat et al. (2024) [43]. In future studies, incorporating k-fold cross-validation will help ensure more reliable generalization and strengthen the validity of the results.

This study included a relatively homogeneous group of participants, which may limit the generalizability of the findings. Prior research has demonstrated that factors such as age, gender, and VR familiarity significantly influence EEG responses [44,45]. For example, older adults often exhibit reduced beta activity and altered connectivity patterns, which could affect classification performance [46]. Additionally, gender differences in alpha and beta power have been observed, particularly during tasks involving spatial navigation and attention [47]. Future studies should aim to include a more diverse participant pool.

Nonetheless, this study highlights the potential of machine learning algorithms, particularly RF, KNN, and SVM, for classifying EEG signals in immersive VR settings. The findings contribute to the growing body of research on analyzing brain cortical responses in immersive environments by systematically comparing multiple classifiers and feature extraction methods. The ability to classify EEG signals with high accuracy could enhance personalized VR experiences, neurofeedback training, and advancements in VR-based brain–computer interfaces (BCIs). Moreover, the results underscore the importance of CSP features in capturing spatial neural patterns, aligning with prior research on the advantages of spatially enhanced EEG features [9,13]. Future work should focus on expanding the feature set and incorporating more diverse VR stimuli, and employing advanced evaluation methods to further enhance the reliability and applicability of these approaches.

## 5. Conclusions

This study successfully applied machine learning algorithms to classify EEG signals recorded during 2D and 3D VR experiences. Two feature extraction methods, PSD and CSP, were evaluated in combination with various classifiers, including SVM [26], KNN [13], RF [7], LR [27], Bayes [15], DT [8], AdaBoost [16], and a voting classifier [17]. The highest classification performance was achieved using CSP features with random forest, which attained an accuracy of 95.02%, a precision of 95.15%, and an F1-score of 94.48%. KNN and SVM also performed well with CSP features, achieving accuracies of 93.16% and 91.39%, respectively. In contrast, models using PSD features generally exhibited lower accuracy, highlighting the superior classification capability of CSP features for this dataset. These findings demonstrate the effectiveness of machine learning algorithms in classifying EEG signals recorded during VR experiences, providing a solid foundation for applying machine learning in VR-related research.

## Figures and Tables

**Table 1:** Machine learning models and parameters.

Classifier	Parameters
SVM	Kernel = RBF, C = 1.0, Gamma = ‘scale’
KNN	k = 5, Distance metric = Euclidean
RF	Number of trees = 100, Maximum depth = None, Criterion = Gini impurity
LR	Solver = ‘lbfgs’, Maximum iterations = 1000, C = 1.0
Bayes	Priors = None, var_smoothing = 1 × 10^−9^
DT	Maximum depth = 5, Criterion = Gini impurity
AdaBoost	Base estimator = Decision Tree (max depth = 5), Number of estimators = 50, Learning rate = 1.0
Vote	Soft voting, combining SVM, KNN, RF, naive Bayes, DT, and AdaBoost

**Table 2:** Performance of different models in binary classification (mean ± standard deviation).

Classifier	Feature Extraction	Accuracy (%)	Precision (%)	Recall (%)	F1-Score (%)
SVM	PSD	81.91 ± 1.12	85.63 ± 1.43	74.69 ± 2.56	79.79 ± 1.67
	CSP	91.39 ± 0.89	91.66 ± 1.02	89.18 ± 1.24	90.40 ± 1.11
KNN	PSD	82.89 ± 1.34	83.02 ± 1.56	80.71 ± 1.87	81.85 ± 1.48
	CSP	93.16 ± 0.76	94.35 ± 0.89	90.36 ± 1.11	92.31 ± 0.98
RF	PSD	83.60 ± 1.21	83.83 ± 1.41	81.39 ± 1.67	82.59 ± 1.38
	CSP	95.02 ± 0.67	95.15 ± 0.82	93.83 ± 0.89	94.48 ± 0.75
LR	PSD	75.83 ± 1.98	77.54 ± 2.14	69.60 ± 2.34	72.04 ± 2.25
	CSP	85.71 ± 1.45	87.62 ± 1.58	81.64 ± 1.79	84.52 ± 1.63
Bayes	PSD	64.35 ± 2.31	62.50 ± 2.54	63.59 ± 2.76	66.73 ± 2.48
	CSP	69.87 ± 1.89	83.86 ± 1.76	45.78 ± 2.32	59.23 ± 2.12
DT	PSD	71.09 ± 1.87	74.15 ± 2.09	60.67 ± 2.56	66.73 ± 2.14
	CSP	82.78 ± 1.12	77.37 ± 1.98	88.65 ± 1.21	82.83 ± 1.34
AdaBoost	PSD	73.70 ± 1.78	72.20 ± 1.93	73.14 ± 2.01	72.67 ± 1.87
	CSP	89.50 ± 0.89	89.46 ± 0.92	88.46 ± 1.12	88.96 ± 0.95
Vote	PSD	81.44 ± 1.10	83.04 ± 1.45	76.86 ± 1.98	79.83 ± 1.52
	CSP	91.96 ± 0.95	96.27 ± 0.68	86.53 ± 1.34	91.15 ± 0.83

## Data Availability

Some of the participant data presented in this study are available upon request from the corresponding author. However, due to privacy concerns, certain participants' data may not be offered.
